# Evaluation of Steady-State and Time-Resolved Fluorescence Spectroscopy as a Method for Assessing the Impact of Photo-Oxidation on Refined Soybean Oils

**DOI:** 10.3390/foods12091862

**Published:** 2023-04-30

**Authors:** Carla Regina Borges Lopes, Lilia Coronato Courrol

**Affiliations:** José de Fillipi Unit, Department of Physics, Institute of Environmental, Chemical and Pharmaceutical Sciences, Campus Diadema, Federal University of São Paulo, Diadema 09972-270, SP, Brazil; lcourrol@unifesp.br

**Keywords:** soybean oil, photo-oxidation, fluorescence spectroscopy, packaging color

## Abstract

The type of material used in packaging, lighting, and storage time can impact food quality during storage. This study aimed to investigate the progress of photosensitized oxidation in refined soybean oil using steady-state and time-resolved fluorescence spectroscopy. The experiment was conducted through accelerated photo-oxidation with Light-Emitting Diode (LED) in samples stored for ten days at room temperature (26.0 ± 2.0 °C) in clear polyethylene terephthalate (PET) packaging of different colors and different transmission spectra in the UV and visible range. Emission spectra were obtained with excitation at 373, 405, and 500 nm, resulting in two main emission peaks: the first with maximum emission between 430 and 555 nm and the second at around 660 nm. Fluorescence decay curves were obtained with excitation at 340 and 405 nm. The results indicated that transparent PET bottles are not effective in protecting soybean oil from photosensitized oxidation under the studied conditions. Strong correlations were observed between fluorescence parameters and peroxide and conjugated diene values, indicators of lipid oxidation progress. Fluorescence spectroscopy has several advantages over traditional methods as it is a simple, fast, low-cost, and low-waste technique.

## 1. Introduction

Refined soybean oils are among the most consumed vegetable oils in the world. From April 2021 to April 2022, about 47.2 million tons were consumed in food products, whether as an ingredient in preparing recipes such as bread, cookies, and cakes or in the frying process [1].

The oxidative stability of vegetable oils is important for food quality and safety, considering that oxidized oils reduce the quality of foods and the ingestion of products resulting from these reactions harms human health [2,3].

The composition rich in polyunsaturated fatty acids (PUFAs) makes soybean oils highly susceptible to oxidation [4]. The PUFAs linoleic and linolenic acid represent 52.5% to 70.0% of their composition [5].

In addition to composition, external factors such as the presence of antioxidants, metals, light, and temperature influence the oxidative stability of vegetable oils [6].

Exposure to light accelerates oil degradation through the process of photo-oxidation, which occurs through the action of photosensitizers such as chlorophyll and heme compounds [7]. These chromophores sensitizer substances in the singlet state absorb energy in the UV or visible region, moving to the excited state. Return to the ground state occurs with the formation of radicals through electron or hydrogen acceptance from the substrate (via type I); or by energy transfer to triplet oxygen (^3^O_2_), converting it to singlet oxygen (^1^O_2_)-via type II. Although it can occur by both pathways, type II is the main mechanism of vegetable oil photo-oxidation, with chlorophyll being the main chromophore sensitizer involved in the process [6].

The action of free radicals does not initiate the type II process and consists of the direct addition of ^1^O_2_ to a double bond carbon, with a change in the position of the double bond and production of a hydroperoxide in the trans configuration. It does not present a measurable induction period, it is unaffected by primary antioxidants, and it is responsible for generating conjugated and non-conjugated hydroperoxides, while autoxidation generates only conjugated hydroperoxides [6].

Proper storage is the main way to prevent or delay autoxidation and photo-oxidation in vegetable oils. Tawfik and Huyghebaert [8] observed significantly higher changes in peroxide value and thiobarbituric acid value in olive, sunflower, and palm oils stored for 60 days in plastic bottles than in glass bottles.

Pristouri et al. [9] reported that temperature and light parameters contribute more significantly to the oxidative stability of extra-virgin olive oil compared with the bottle headspace and oxygen transmission rate of the packaging material. The authors reported increased oxidation indicators in the oil stored in transparent polyethylene terephthalate (PET) packaging, exceeding the acidity limit after six months. The limit was not exceeded even after 12 months of storage for the oil packaged in PET covered with aluminum.

According to the EN-12464-1 Standard of the European Commission [10], supermarkets must have a minimum illuminance of 300 lux. In Brazil, the ABNT NBR ISO/CIE 8995-1 Standard [11] determines an ideal illuminance of 500 lux for these establishments.

The compact fluorescent lamp (CFL) has been widely used in recent decades due to its energy efficiency and greater durability. However, Light-Emitting Diode (LED) is currently the predominant technology due to its lower energy consumption [12]. White LED lamps emit throughout the visible spectrum with maximum emissions at ~450 nm and between 500 and 650 nm [13]. Chlorophyll, the primary photosensitizing substance in vegetable oils, absorbs light in these regions [14].

Optimizing methods to evaluate the oxidative status is as important as ensuring proper storage of vegetable oils. Several methods are described for evaluating lipid oxidation, ranging from organoleptic evaluations to physical, chemical, and physicochemical methods [5].

The main limitation is that established methods generally provide information on specific stages of the oxidation process and some are more applicable to specific lipid systems than others [15,16].

Peroxide value (PV), acidity value (AV), and conjugated dienes (CD) are commonly used indicators of oxidative processes in vegetable oils. These methods have disadvantages, such as measuring products present in specific stages of oxidation, as well as high consumption of solvents and reagents, resulting in potentially toxic waste generation [16].

In this context, fluorescence spectroscopy has been widely studied as an alternative for food quality control. The technique has advantages such as sensitivity to detect a trace of fluorescent compounds, specificity, immediate results, non-destructiveness for food samples, ease of use with low-cost equipment, and minimal training for application and analysis of results [17,18].

It should be noted that most recent studies involving fluorescence in the analysis of vegetable oils are related to determining the origin or adulteration in extra-virgin olive oil. Ali et al. [19] used fluorescence spectroscopy with excitation at 350 nm to analyze the adulteration of olive oil with sunflower oil, identifying an increase in the region between 441 and 489 nm and a reduction in the chlorophyll emission peak with increasing sunflower oil concentration. Al Riza et al. [20] described front-face fluorescence to identify specific characteristics of olive oils, allowing authentication of the geographical origin and type of oil, using an emission-excitation matrix, and applying chemometric methods.

Several publications concerning the effect of packaging parameters can be cited for treating the materials with high nutritional and commercial value and the high rate of counterfeiting and adulteration to which they are subjected [21,22,23,24].

Regarding the use of fluorescence spectroscopy in the characterization and determination of oxidative state in crude and refined oils, Poulli et al. [25] described the study of synchronous fluorescence spectra in monitoring the thermal stress of vegetable oils, indicating the spectroscopic characteristics in the range of 320 to 520 nm ideal for the analysis of soybean, corn, and sunflower oils. Hao et al. [26] investigated the potential of laser-induced fluorescence spectroscopy combined with chemometric techniques to determine the addition of residual frying oil (therm-oxidized) to fresh oil.

In a previous study, we described the use of fluorescence to determine the progression of oxidation as a function of heating conventional and organic refined soybean oils [27].

In this study, the potential of fluorescence spectroscopy was investigated to indicate the progress of photosensitized oxidation through correlation analysis between steady-state and time-resolved fluorescence parameters with peroxide (PV) and conjugated diene (CD) values. Fluorescence spectroscopy can be used as an alternative to traditional methods to qualitatively determine the advancement of oxidation in vegetable oils exposed to UV and visible radiation.

## 2. Materials and Methods

### 2.1. Chemicals and Oils

Isopropanol PA-ACS (C_3_H_8_O) was acquired from LabSynth© Diadema, SP Brazil LTDA.

Refined soybean oil genetically modified with *Agrobacterium tumefaciens* and *Streptomyces viridochomogenes* and supplemented with citric acid antioxidants was purchased from the local market.

### 2.2. Accelerated Photo-Oxidation in a Light Chamber

**Figure 1:**
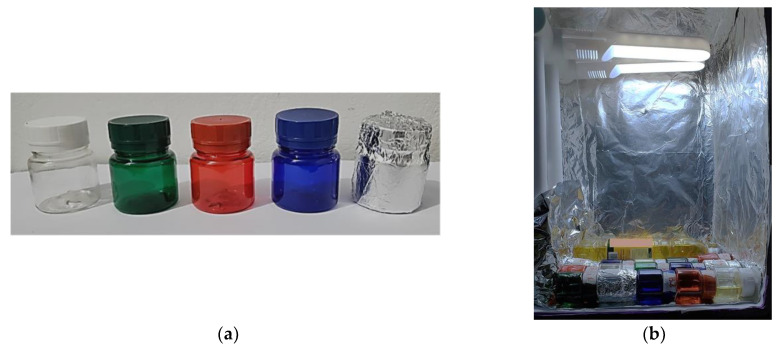
(**a**) Polyethylene terephthalate (PET) bottles used in the experiment. (**b**) Samples in the accelerated photo-oxidation chamber.

In triplicate, 30 mL aliquots of the studied oil were placed in clear PET bottles with a capacity of 50 mL, 5.3 cm in height and 4.5 cm in diameter, in colorless, green, red, blue, and a colorless bottle wholly covered with aluminum foil (Figure 1a).

To determine the transmission spectra of the bottles between 200 and 800 nm, a spectrophotometer UV 2600 Plus manufactured by Shimadzu©, Kyoto, Japan© was used. The spectra are presented in Figure S1.

Samples can be identified according to Table 1.

The bottles were kept for ten days at room temperature (26.0 ± 2.0 °C) in an accelerated photo-oxidation chamber: a rectangular box with dimensions (cm): 22 × 33 × 44 (H × W × D) covered with aluminum paper, containing 2 LED lamps with a cold white color temperature of 5000 K, 4 Watts of power, and 320 lumens of luminous efficiency each. The bottles were positioned at the bottom of the box at 32.5 cm from the lamps in a horizontal position to prevent the light from being blocked by the caps made of opaque plastic material (Figure 1b). The sample’s surface area in the bottle was 24 cm^2^, and the headspace was 20 mL.

The average illuminance inside the chamber was 8815 lux, calculated from Equation (1) [28]. Luminance measuring software for Android was used to determine variations in light incidence in the chamber area. Measurements were taken from three different pieces of software, and the averages were considered. Variations of up to 20% in illuminance were recorded at different points in the chamber. The bottles were moved to another position every 48 h. At each position change, the light incidence on each bottle was measured. At the end of the experiment, the average illuminance on each bottle was 9800 lux, equivalent to approximately 20 times the typical average luminous incidence in supermarkets [10].

Lux = lumens/m^2^ (1)

### 2.3. Oils Analysis

Peroxide value (PV): Peroxides are all substances that can oxidize potassium iodide (KI) and are expressed in milliequivalents of active oxygen per kg of oil (meq O_2_/kg). The HI83730-01 Photometer for Peroxide Content in Oils from Hanna Instruments©, Woonsocket, Rhode Island, USA [29] was used to determine PV.

The equipment adapts the method set out in Regulation CEE 2568/91 and its amendments [29] to measure peroxide values below 25.0 meq O_2_/kg.

Sample preparation and measurement were performed according to the protocol and manufacturers. Briefly, the sample was added to the ready-to-use reagent provided by the manufacturer composed of chloroform, acetic acid, and potassium iodide. In the presence of peroxides, the solution turned yellow. The darker the color, the higher the concentration of peroxides. This color change is then analyzed colorimetrically according to the Lambert–Beer Law, using a silicon photocell with an interference filter at 466 nm.

Equipment specifications: Source: Tungsten lamp. Resolution 0.5 meq O_2_/kg. Precision ± 0.5 meq O_2_/kg. Detection limit from 0.0 to 25.0 meq O_2_/kg.

Conjugated dienes (CD): The method used was adapted from AOCS-Ch 5-91 [30]. Briefly, an aliquot of approximately 0.03 ± 0.005 g of oil was diluted in isopropanol at a concentration of 1:1000 (w/w), and the absorbance at the wavelength of 232 nm, related to the conjugated diene absorption region [31] was measured. Spectra between 200 and 320 nm were obtained using the spectrophotometer UV 2600 Plus manufactured byShimadzu©, Kyoto, Japan and are available in Figure S2. Results are expressed in terms of specific extinction (E) or absorptivity calculated from Equation (2):

(2) $$\frac{A\lambda }{c\times l}=K\lambda$$

where *Kλ* = specific extinction or absorptivity at wavelength *λ*; *Aλ* = absorbance measured at wavelength *λ*; *c* = concentration of the solution in g/100 mL; *l* = optical path length of the cuvette in cm.

### 2.4. Fluorescence Measurements

#### 2.4.1. Steady-State

Fluorescence spectra were obtained using the fluorometer Fluorolog 3 manufactured by Horiba Jobin Yvon© Kyoto, Japan. Measurements were performed on samples at room temperature without any preparation. Approximately 2.5 ± 0.5 mL of each sample was placed in a quartz cuvette with four polished sides with dimensions of 10 mm × 10 mm (width × depth), with a capacity of 3.5 mL. Fluorescence was measured at a 90° angle to the excitation light. The region between 300 and 800 nm was analyzed, with excitation wavelengths (*λ*_Ex_.) applied for each measurement, with intervals of Δ = 20 nm, between 200 and 500 nm.

The spectra were processed using the software Origin 8.5©. The spectral characteristics of the main emission peaks were analyzed: integrated area under the band, wavelength with maximum emission (*λ*_Em.Max._) in nanometers (nm), and emission intensity (I) expressed in photon counts per second (CPS).

#### 2.4.2. Time-Resolved

The oil samples were diluted in isopropanol at a concentration of 1:100 (w/w) for analysis. The fluorescent decay time was obtained using a system composed of pulsed sources of picoseconds (ps) manufactured by PicoQuant© Berlin, Germany, with the following specifications:

LDH series diode laser. Wavelength: 405 nm. Spectral width: 2 to 8 nm. The repetition rate of 80 MHz.

PLS series pulsed LED. Wavelength: 340 nm. Spectral width: <10 nm. Pulse width (typical): 800 ps. The maximum repetition rate of 10 MHz.

The power levels and repetition rate were adjusted using the corresponding driver from the PDL series. The signal was detected with a PMA photomultiplier. Bandpass (BP) filters manufactured by ThorLabs© New Jersey, USAwere used to separate the emission signals. The transmittance spectra of the BP filters used are available in Figure S3.

The fluorescence decay curves were fit with a single, double, or triple exponential function, considering the best fit based on the smallest Chi-squared (χ^2^) and a coefficient of determination (R^2^) closest to 1. The mean fluorescence lifetime (*τ*) was calculated using Equation (3):

(3) $$\langle \tau \rangle =\frac{\Sigma A_{i}\tau_{i}^{2}}{\Sigma A_{i}\tau_{i}}$$

where *τ_i_* are the lifetime components and *A_i_* their relative amplitudes. The fluorescence decay time fits are available in Supplementary Worksheet S1.

### 2.5. Correlation Analysis between Fluorescence Parameters and PV and CD

The Pearson linear correlation coefficient was calculated using Microsoft Excel 365© software. The Pearson correlation evaluates the linear relationship between two variables; that is, it assesses whether a change in one variable is associated with a proportional change in the other variable.

The correlation coefficients can range from r = −1 to r = 1. Negative values indicate a negative correlation, where one variable increases as the other decreases. Positive values indicate that when one variable increase or decreases, the other presents the same behavior. The closer to r = −1 or r = 1, the stronger the correlation. Values of r = −1 or r = 1 indicate a perfect linear relationship.

## 3. Results and Discussion

The transmittance spectra of the PET bottles used to store the samples during the experiment are available in Figure S1. The clear bottle allows the passage of 39% to 47% of the light from ~320 nm. The bottle covered with aluminum foil blocks ≥99% of light across the entire spectrum studied. Considering the colored bottles, the green one offers greater protection in the UV and visible region up to 460 nm and ≥650 nm, while the red bottle blocks light more efficiently in the region between 460 and 575 nm. The blue bottle allows higher light transmission at 458 nm, with a maximum transmittance of 52.49% in this region, and blocks ≥99.9% between 580 and 650 nm.

### 3.1. Analysis of the Oxidation State of the Samples-PV and CD

**Figure 2:**
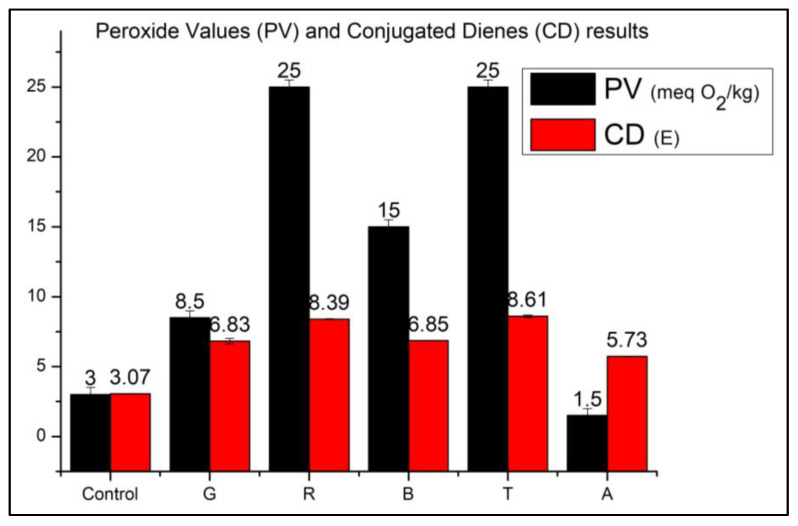
Peroxide values (PV) and conjugated dienes (CD) results.

Figure 2 presents the results related to PV and CD measurements. At the beginning of the experiment, the recorded PV was 3.0 ± 0.5 meq O_2_/kg. According to the standards described in the Codex Alimentarius, refined oils with peroxide values below 10 meq O_2_/kg are in optimal storage conditions [5].

The increase in PV is attributed to the formation of hydroperoxides, which represent primary oxidation compounds. Thus, PV can be considered an indicator of the initial phase of oxidative modifications. After the experiment, among the translucent colored bottles, only sample G remained below the limit of 10 meq O_2_/kg. The highest PV was recorded for sample T, which exceeded the equipment limit of 25.0 meq O_2_/kg. The registered peroxide value is T>R>B>G>A in decreasing order.

In the present study, PV was obtained using a portable photometer for peroxide detection, as described in Section 2. The equipment returns the PV based on a colorimetric analysis, where the higher the concentration, the darker the sample color. Images of the samples obtained immediately after analysis are available in Figure S4. Samples R and T have a darker yellow color than the others, while sample A shows little change compared to the original oil color.

The similar coloration among the samples can lead to errors. Sample A had a lower value than the control value. Some authors point to problems arising from the lack of precision in determining the peroxide value [15,16]. The presence of oxygen in the reaction medium can interfere with the process, oxidizing I^−^ and generating I_2_, which can overestimate PV. Another problematic factor is the endpoint of the titration, which can be challenging to visualize in samples with low peroxide content, even in the presence of an indicator [32].

In the CD analysis, an increase in value was recorded in all samples after the period of stay in the photo-oxidation chamber (Figure 2), following the decreasing order: T>R>B>G>A>Control. Although there is no established limit for the CD value specifically in soybean oils, the Codex Alimentarius sets a specific extinction limit at 232 nm of E = 3.5 for extra-virgin olive oils, E = 6.0 for refined olive pomace oil, and E = 5.5 for a mixture of these two oils [33].

Lipid oxidation results in the formation of conjugated compounds, which involves removing allylic and diallylic hydrogens from unsaturated fatty acid molecules, leading to the displacement of the double bond and the formation of conjugated dienes, which makes the molecule more stable [34]. As a result, the absorption energy decreases, leading to a bathochromic shift in the absorption wavelength and an increase in absorption intensity. In their pure state, vegetable oils absorb at 210 nm due to the isolated carbon–carbon double bonds in oleic, linoleic, and linolenic acids. When oxidized, fatty acids present conjugated unsaturations that show intense absorption in the 230 to 270 nm [35].

The formation of conjugated dienes is related to the formation of peroxides. Thus, the correlation analysis between the values is an alternative to minimize intrinsic errors in both analyses and increase the reliability of determining the oxidative state of the oil [31]. In the present study, the analysis shows a strong positive correlation between the results, with r = 0.85. Although there is a strong positive correlation, we observe that the higher the PV, the more significant the difference compared to the CD value. The formation of non-conjugated hydroperoxides, which only occurs in photosensitized oxidation reactions and is not considered in this analysis, may be responsible for this difference in more advanced stages of oxidation [31].

Based on these results, we can indicate that in relation to the oxidative state, the studied samples are presented in the following order, following in increasing order: A<G<B<R<T.

Considering the percentage of light protection in the bottles used in the experiment and the advance of the oxidative state of the samples, it is possible to affirm that the colorless bottle is not adequate for packing soybean oil due to photosensitized oxidation. The high PV and CD recorded for sample T, stored under these conditions (Figure 2), indicate that the sample reached an advanced stage of oxidation after ten days of exposure to light under the studied conditions.

The red bottle, which offers greater protection in the region between 450 and 550 nm compared to the others, was also ineffective in protecting the sample (PV = 25.0). The sample stored in the green bottle was the only one to present PV < 10.0 meq O_2_/kg (Figure 2). The green bottle has lower light transmittance in the region between 320 and 460 and higher between 550 and 650 nm compared to the blue bottle. This result was already predicted, considering that energy is inversely proportional to wavelength. Thus, the shorter the wavelength, the greater the incident radiation energy and the higher the oxidation rate [36].

### 3.2. Fluorescence Analysis

#### 3.2.1. Steady-State

**Figure 3:**
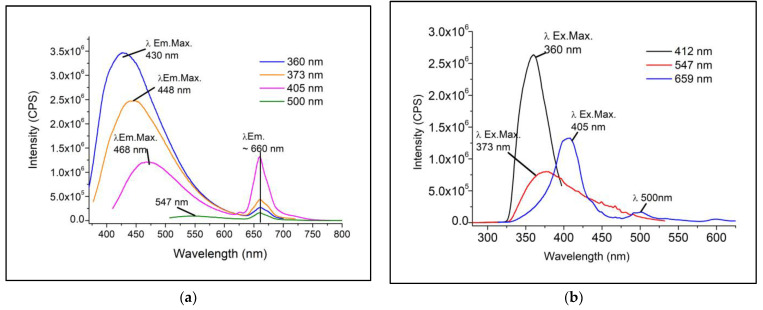
Emission and excitation spectra of soybean oil studied before exposure to accelerated photo-oxidation. (**a**) Emission spectra obtained with excitation (*λ*_Ex_.) at 360, 373, 405 and 500 nm. (**b**) Excitation spectra obtained with fixed emission at 412 and 547 and 659 nm.

The excitation and emission spectra recorded before the exposure period in the photo-oxidation chamber are shown in Figure 3. Figure 3a presents the emission spectra obtained with excitation at 360, 373, 405, and 500 nm. Two main emission bands were observed: the first between 400–600 nm with maximum emission between 430–550 nm. The fluorescence in this region is related to several fluorescent compounds present in vegetable oils, such as free fatty acids, vitamins, phenolic compounds, and oxidation products; the second between 650–750 nm with a maximum emission at ~660 nm is related to the fluorescence of pigments such as chlorophylls and pheophytins [37,38,39,40]. 

The excitation spectra obtained with fixed emission at 412, 547, and 659 nm are shown in Figure 3b. It can be observed that bands around 350–380 nm correspond to the highest absorbers for the emissions of free fatty acids, vitamins, phenolic compounds, and oxidation products, and the bands around 405 and 500 nm are related to the absorption of carotenoids, chlorophylls, and pheophytin.

Considering the emission band registered between 400 and 600 nm, similar spectra have been recorded by other authors in refined vegetable oils, which relate this region to the emission of tocopherols (vitamin E), flavins, and their derivatives (vitamins from the B complex), and oxidation products such as hydroperoxides and free fatty acids [24,28,38,41,42,43,44,45,46]. 

Analyzing the emission region related to chlorophyll fluorescence (*λ*_Em.Max_. ~660 nm), the studied oil exhibits intense fluorescence when excited in the visible region (Figure 3b), which is also identified when excited in the UV region, with lower intensity (Figure 3a).

We verified an intense chlorophyll band in the studied sample, probably due to extraction and refining mechanisms. A factor that can interfere with chlorophyll concentration in soybean oil is the percentage of green/immature grains [47]. 

To confirm that this is not a peculiarity of the studied batch of oil, we compared the emission spectra with those obtained from three other batches of the same brand and six more samples, including three batches of soybean oil produced by two other manufacturers. The results were similar, with an average of 1.85 × 10^6^ CPS (σ = 6.99 × 10^5^ CPS) in the emission intensity in this region (results not presented). In previous studies, we also identified the emission band at ~660 nm in refined soybean oils [27]. 

Another justification for the high chlorophyll concentration may be the bleaching process’s inefficiency, a stage of oil processing that removes pigments. This process is based on the adsorption of pigments, where bleaching clays, special silica, activated carbon, or a mixture of these materials can be used. To reduce production costs, in some cases, the method used by the industry may not present a high adsorption capacity for the pigments [48]. 

This result is significant, considering that these oils are sold in transparent PET packaging and the high chlorophyll concentration can lead to photosensitized oxidation even before the end consumer opens the original bottles.

**Figure 4:**
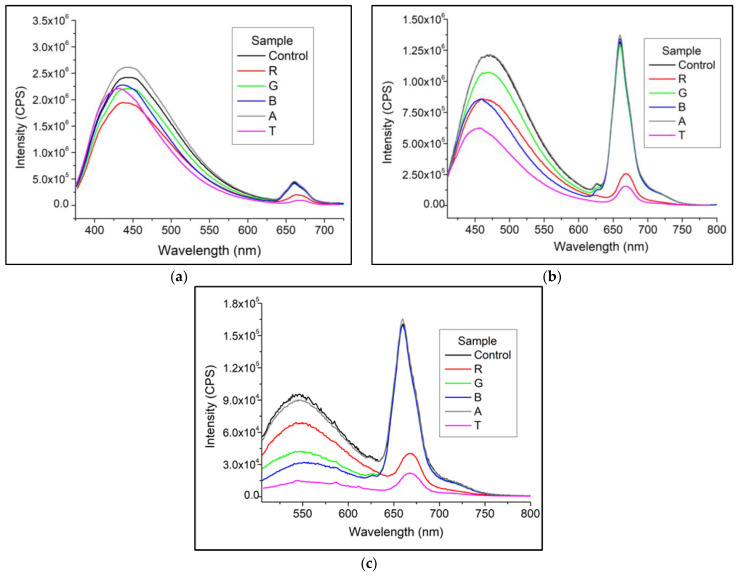
Emission spectra before and after accelerated photo-oxidation: (**a**) *λ*_Ex_. 373 nm, (**b**) *λ*_Ex_ 405 nm, (**c**) *λ*_Ex_ 500 nm.

The fluorescence spectra of the samples after the period in the photo-oxidation chamber obtained with excitation at 375, 405, and 500 nm are presented in Figure 4a–c, respectively. The values referring to the fluorescence parameters recorded in all conditions studied are summarized in Table S1.

In all studied conditions, a quenching of chlorophyll fluorescence (*λ*_Em.Max_. ~660 nm) is observed in samples R and T. The most remarkable differences were recorded when exciting the samples at 405 nm: I = 1.22 × 10^6^ CPS before the experiment (Control); I = 2.06 × 10^5^ ± 8.79 × 10^4^ CPS for sample R and I = 1.35 × 10^5^ ± 1.13 × 10^3^ CPS for sample T (Figure 4a).

Bianchi et al. [14] described the effect of chlorophyll on the photo-oxidation of soybean oils. In the study, known chlorophyll concentrations were added to pure oil, and the samples were exposed to broad-spectrum light and fixed wavelengths of 430 and 660 nm for 4 h. The authors recorded a significant increase in PV and malondialdehyde and chlorophyll degradation to undetectable concentrations in samples exposed to light at 430 nm and broad spectrum. For samples kept in the dark, the authors report no significant differences in chlorophyll concentration or oil oxidation indicators studied.

The authors’ report is consistent with what was observed in the present study, considering that commercial LED lamps, such as those used in this experiment, emit throughout the visible region with a maximum emission at ~450 nm [13]. Thus, this pigment was degraded in samples T and R stored in bottles that offer less protection from light in this region (~450 nm).

In photosensitized oxidation, energy transfer occurs between chlorophyll and ^3^O_2_, which is converted to ^1^O_2_. Energy transfer between the excited species and other molecules results in dynamic suppression of fluorescence. This effect decreases fluorescence intensity, quantum yield, and lifetime [49].

Regarding the first emission region (~400–600 nm), sample excitation in the UV region (*λ*_Ex_./*λ*_EmMax_. 373/448 nm—Figure 4a) resulted in a slight increase in emission intensity of bottle A and quenching in the others. However, the hypsochromic shift of the emission peak (*λ*_Em.Max_) is the spectral characteristic that changed the most considering the oxidative state of the samples: while the control sample emits around 449 nm, the sample irradiated in bottle T emits ~432 nm.

As previously reported, this region is related to the emission of various fluorescent compounds in the oil, and signal overlap makes their identification difficult. Some of the main fluorophores that emit in this region are vitamins and oxidation products such as hydroperoxides and free fatty acids [38,41]. The position of the emission peak is a spectral characteristic related to the nature of the fluorophore. Thus, changes in this characteristic may indicate chemical changes in the studied material [36].

Photosensitized oxidation increases the concentration of hydroperoxides and conjugated and unconjugated dienes formed from the oxidation of ^1^O_2_. In later stages, these compounds are degraded, giving rise to secondary oxidation products and altering the substrate’s composition. 2-Heptenal and 2-butenal are some of the decomposition products of hydroperoxides resulting from the oxidation of linoleic and linolenic acid by ^1^O_2_ [6].

The increase in the concentration of these compounds and the degradation of unsaturated fatty acids and vitamins naturally present in the oil due to the advancement of photo-sensitized oxidation reactions alters the oil’s composition and, consequently, the characteristics of the emission spectra. In a previous study on the thermo-oxidation of soybean oils, we identified an increase in fluorescence at ~450 nm (ex. 400 nm) after the first heating, followed by suppression after repeated heating [27].

When excited at 500 nm (Figure 4c), the emission intensity at ~550 nm of sample R was higher than B and G (3.64 × 10^4^ ± 2.51 × 10^3^ CPS; 1.63 × 10^4^ ± 1.03 × 10^3^ CPS; and 1.86 × 10^4^ ± 1.22 × 10^3^ CPS, respectively). However, considering the PV, CD (Figure 2), and the suppression of chlorophyll fluorescence (Figure 4), it is correct to assume that samples B and G are in a less advanced oxidation stage than R.

This excitation/emission region (500/550 nm) can be related to riboflavins (vitamin B-derived), and carotenoids, highly oxidizable compounds naturally present in vegetable oils [48,50,51]. The red bottle used in this study offers greater protection from light in the absorption region around 500 nm compared to the others (Figure S1), which may be responsible for reducing its degradation during light exposure.

Similar to chlorophylls, carotenoids can be partially removed during refining [48]. These pigments act as antioxidants in the photosensitized oxidation process by extinguishing the triplet sensitizer or ^1^O_2_ in an energy transfer process [51]

#### 3.2.2. Time-Resolved

**Figure 5:**
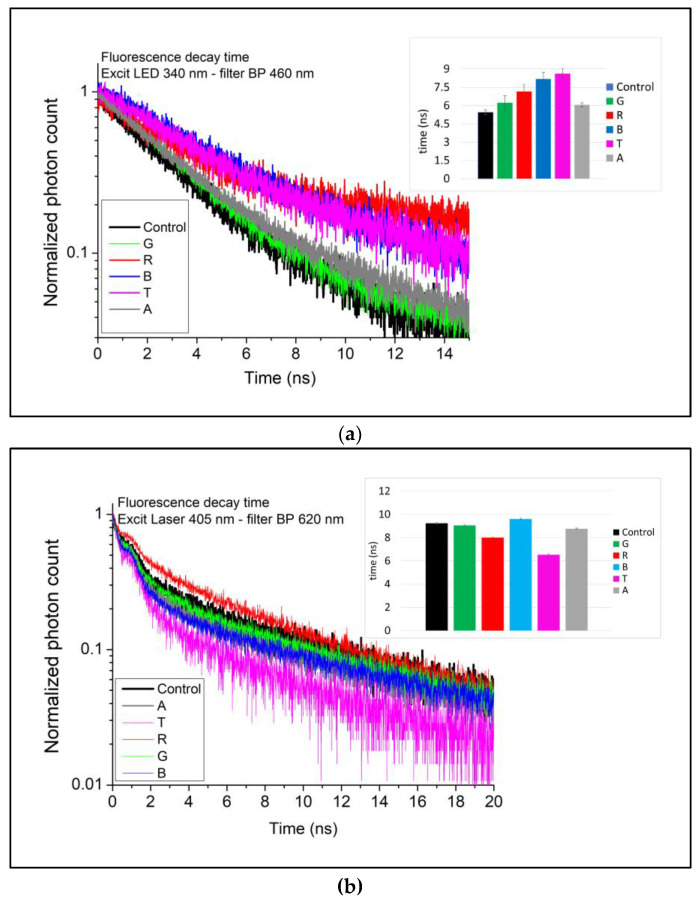
Fluorescent decay curves of samples before and after accelerated photo-oxidation. The inset represents the lifetimes (*τ*) for each sample. (**a**) Excitation: LED 340 nm/BP filter 350 nm; (**b**) Laser 405 nm/BP filter 620 nm.

The fluorescent decay curves are shown in Figure 5. The double exponential model provided the best fit in curves obtained under excitation with 340 nm LED and an emission filter BP 460 nm (Figure 5a). A longer lifetime τ was observed in all samples compared to that recorded before the experiment (*τ* = 5.44 ns), with sample T having the longest fluorescent decay time (*τ* = 8.62 ns). Fluorescence in this region may originate from various components, primarily advanced lipid oxidation products such as aldehydes [52]. An increase in malondialdehyde has been related to an increase in fluorescence lifetime in this region [53]. A strong negative correlation (r = −0.91) existed between lifetime and emission intensity at ~460 nm.

The double exponential model also provided the best fit for the fluorescent decay curves obtained with excitation at 405 nm and a BP620 emission filter (Figure 5b). Under these conditions, the lifetime recorded before accelerated photo-oxidation was *τ* = 9.24 ns, with reductions in the photo-oxidized samples. The shortest lifetime was recorded in sample T: *τ* = 6.2 ns. This excitation/emission region is related to the lifetime of chlorophylls and can be compared to the fluorescence quenching at ~660 nm in the emission spectra obtained with excitation at 405 nm (Figure 4a). There was a strong positive correlation (r = 0.89) between lifetime and emission intensity under the cited conditions.

Fluorescence lifetime refers to the time a fluorophore takes to return from the excited state to the ground state. Electron loss (energy transfer) leads to a reduction in decay time [36].

As described in the introduction, photosensitized oxidation (Type II) occurs through the absorption of radiation by a photosensitive compound that goes from the ground state to the excited state, and its return to the ground state involves energy transfer to ^3^O_2_, which is converted to ^1^O_2_, which reacts directly with the double bonds of fatty acids to form conjugated and non-conjugated hydroperoxides [6]. The fluorescence quenching at ~660 nm and a reduction in chlorophyll lifetime indicate energy transfer, confirming the occurrence of type II photo-oxidation.

In this case, it is possible that triplet chlorophyll reacted with molecular oxygen to produce ^1^O_2_. The formed ^1^O_2_ can then directly attack the chlorophyll or be transformed into another reactive oxygen species [54].

### 3.3. Correlation between Fluorescence Parameters and PV/CD

The correlation coefficients obtained between PV, CD, and fluorescence parameters are available in Table 2. Strong or moderate correlations were recorded for at least one of the parameters in all conditions studied. These results indicate that the changes observed in the emission spectra and fluorescence lifetime are directly related to the oxidative changes in the soybean oil studied.

Considering the steady-state fluorescence parameters, the spectra obtained with excitation at 373 and 405 nm returned the best results. The strongest correlation with lipid oxidation markers was recorded with excitation at 340 nm with a BP460 filter for the lifetime. Correlations with PV were more robust than with CD. This is possible because CD analysis does not measure non-conjugated dienes resulting from ^1^O_2_ oxidation.

**Figure 6:**
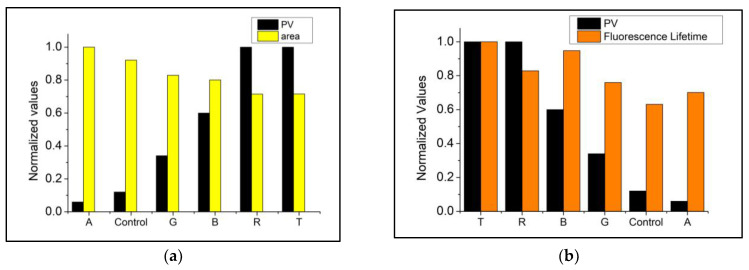
Comparison between normalized PV and fluorescence parameters. (**a**) Integrated area *λ*_Ex_. 373 nm *λ*._Em.Max._ ~448 nm; (**b**) Lifetime *λ*_Ex_. 340 nm, BP 460 nm filter.

The fluorescence parameters with the strongest correlations and PV are presented in Figure 6. Considering the significant differences in the values scales, they were normalized to enable comparison using a standard scale without distorting the interval differences or losing information.

The standard PET packaging used for the commercialization of vegetable oils in Brazil, despite representing an excellent barrier to oxygen and odors, allows up to 80% of light transmission from 310 nm [55] and may not be suitable for maintaining the oxidative stability of soybean oil during storage, which can result in degradation even before the final consumer opens the bottles. 

Considering small commercial establishments or street food stalls, where oil bottles are exposed for commercialization or used for food preparation, the direct sunlight on sunny days can exceed 100,000 lux, equivalent to more than ten times the light incidence used in the experiment conducted in the present study. Without direct sunlight, on a sunny day, the light incidence can reach 2000 lux indoors and 32,000 lux outdoors [28].

Some alternatives have been adopted, such as adding UV absorbers to the polymer used for packaging manufacturing. These usually are compounds derived from benzophenone or benzotriazole that act by absorbing UV radiation, intending to delay photo-oxidation. However, these compounds effectively block light only at wavelengths <400 nm, still allowing visible light transmission in regions of photosensitizer absorption, such as chlorophyll (~450 nm). In addition, these compounds must be controlled because there is the possibility of migration to the stored food [55].

Based on this information and considering the results observed in the present study, it is important for the food industry to evaluate the packaging used to commercialize vegetable oils. We believe the ideal solution would be a physical barrier completely blocking UV and visible light incidence.

A viable alternative would be the addition of an opaque plastic wrapper to translucent PET, avoiding the industry’s loss with the disposal of already acquired and stocked packaging. Gradually, translucent packaging could be replaced by opaque packaging capable of protecting vegetable oils from light. 

In the medium and long term, we estimate that this action could result in increased yields for vegetable oil manufacturers with an increase in product shelf life. We also believe that the commercialization of oils in opaque packaging would not diminish consumer attractiveness, considering that vegetable oils are essential products and were sold for decades in metallic packaging that did not allow their visualization.

Steady-state fluorescence spectroscopy presented advantages over traditional chemical methods, such as eliminating dilution and sample preparation procedures, avoiding the handling of potentially toxic reagents and waste generation, and reducing the time and cost of analysis, in addition to being non-destructive. The technique can be used as an alternative in screening soybean oil samples and can qualitatively indicate advanced stages of oxidation in soybean oils. The strong correlation between the parameters of emission intensity and fluorescent decay can help to understand the mechanisms involved and contribute to the availability of data on fluorescence lifetime in the specialized literature.

Additional experiments employing the more specific methods to determine lipid peroxidation products LOPs such as aldehydes could be monitored, making the fluorescence a quantitative method to predict the oxidation indicators in the routine of a food industry.

The analysis of oils extracted from other raw materials such as corn, sunflower, canola, among others, is also necessary because differences in the fatty acids composition and in the chlorophyll and carotenoids concentration can result in fluorescence changes different from those recorded for soybean oil.

## 4. Conclusions

Soybean oils kept in a photo-oxidation chamber with LED lamps and an average light incidence of 9800 lux for 10 days showed an increase in PV and CD. The sample packaged in a colorless bottle, similar to that used in soybean oils marketing, showed high values of PV and CD, indicating that this packaging type is unsuitable for storage. Among the clean packages analyzed, the green bottle, which offers greater UV radiation protection, was the only one to keep the sample with a peroxide index below the limit of 10 meq O_2_/kg. However, there was an increase in the oxidative state in this packaging due to the degradation of riboflavins and carotenoids, pointed by emission intensity reduction around 550 nm (λ_Exc_ 500 nm). The bottle covered in aluminum foil effectively protected the sample, resulting in low PV and CD values. In this way, we consider it ideal for replacing soybean oil packaging with others produced in a material capable of protecting it from UV and visible light. Changes in the λ_Em.Max_. and in the integrated area of the band of the first emission region of the samples excited at 373 nm (λ_Em.Max_. ~448 nm), as well as the reduction in the lifetime of fluorescence with excitation at 340 nm and emission filter BP 460 nm showed strong negative correlation with PV and CD. Fluorescence spectra can be used alternatively to indicate an advanced stage of oxidation based on the comparison with spectra obtained from reference oils. Its use can significantly reduce time and costs by controlling the progress of oxidation in soybean oils in the food industry.

## Figures and Tables

**Table 1:** Packaging materials.

Sample	Label
Soybean oil before the photo-oxidation experiment.	Control
Green bottle	G
Red bottle	R
Blue bottle	B
Colorless bottle	T
Bottle covered with aluminum foil	A

**Table 2:** Correlation coefficients between fluorescence parameters at indicated excitation/emission wavelengts (***λ*_Ex_./*λ*_Em.Max._**), Peroxide Value (PV), and Conjugated dienes (CD).

*λ*_Ex_./*λ*_Em.Max._ (nm)	373/~448			373/~660		
Marker	Area	*λ*_Em.Max._	Intensity	Area	*λ*_Em.Max._	Intensity
**PV**	−0.96	−0.95	−0.88	−0.85	0.75	−0.88
**CD**	−0.79	−0.91	−0.71	−0.68	0.66	−0.72
***λ*_Ex_./*λ*_Em.Max._ (nm)**	**405/~468**			**405/~659**		
**Marker**	**Area**	***λ*_Em.Max._**	**Intensity**	**Area**	***λ*_Em.Max._**	**Intensity**
**PV**	−0.89	−0.87	−0.93	−0.87	0.89	−0.88
**CD**	−0.79	−0.76	−0.82	−0.70	0.73	−0.72
***λ*_Ex_./*λ*_Em.Max._ (nm)**	**500/~547**			**500/~659**		
**Marker**	**Area**	***λ*_Em.Max._**	**Intensity**	**Area**	***λ*_Em.Max._**	**Intensity**
**PV**	−0.52	0.28	−0.59	−0.83	0.87	−0.86
**CD**	−0.62	0.04	−0.68	−0.65	0.69	−0.69
***λ*_Ex_./*λ*_Em.Max_ (nm)**	**340/460**			**405/620**		
**Marker**	**Fluorescence lifetime**			**Fluorescence lifetime**		
**PV**	0.81			−0.69		
**CD**	0.80			−0.64		

## Data Availability

Data are contained within the article or Supplementary Material.

## Supplementary Materials

The following supporting information can be downloaded at: https://www.mdpi.com/article/10.3390/foods12091862/s1, Figure S1: Transmittance spectra of PET bottles used to store samples during the experiment; Figure S2: UV absorbance spectra of samples for determination of conjugated diene values; Figure S3: Transmittance spectra of Bandpass (BP) filters used for separation of emission signals in fluorescence lifetime measurements. (a) BP460; (b) BP620; Figure S4: Samples after the reaction time with the ready reagent supplied by the manufacturer for analysis of peroxides. (N) Control; (b) Sample R; (c) Sample G; (d) Sample B; (e) Sample T; (f) Sample A; (g) from left to right samples: R, G, B, T, A; Worksheet S1: Fluorescence decay time fits; Table S1: Steady-state fluorescence parameters.
