# Supplementary material for: Evaluation of Steady-State and Time-Resolved Fluorescence Spectroscopy as a Method for Assessing the Impact of Photo-Oxidation on Refined Soybean Oils
Source: Foods. 2023 Apr 30;12(9):1862. doi: 10.3390/foods12091862 (PMC10178558; doi:10.3390/foods12091862)

# “Evaluation of steady-state and time-resolved fluorescence spectroscopy as a method for assessing the impact of photo-oxidation on refined soybean oils”

Carla Regina Borges Lopes and Lilia Coronato Courrol

## Supplementary Material

Figure S1 - Transmittance spectrum of PET bottles used to store samples during the experiment

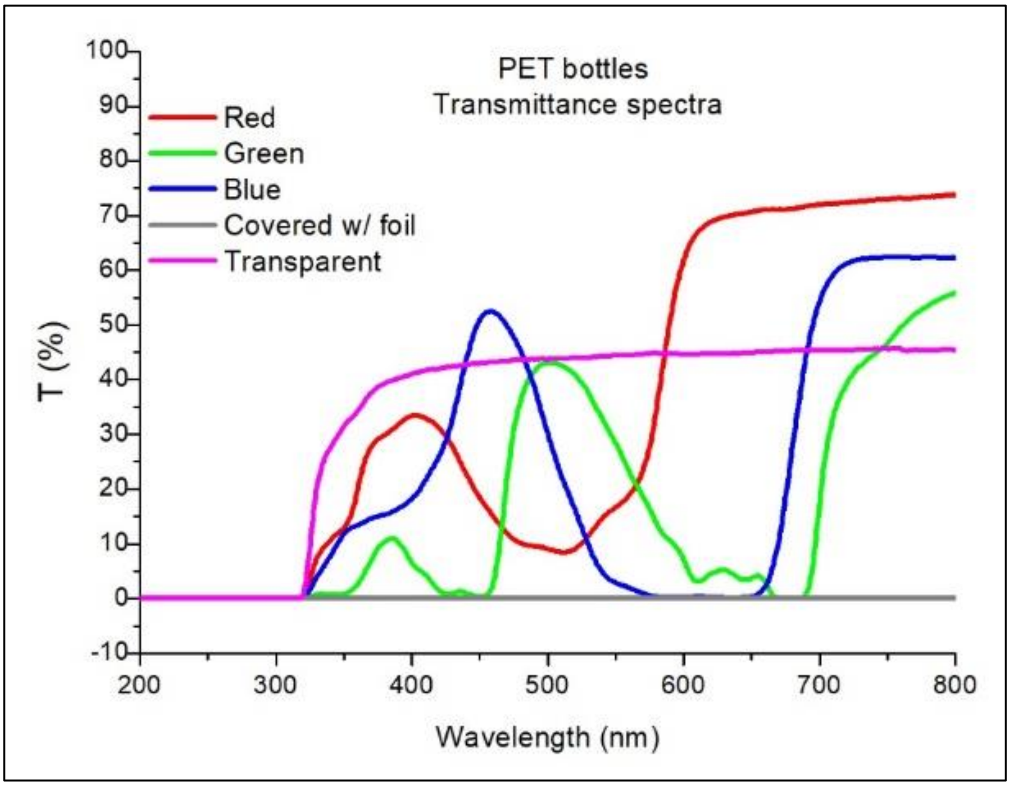

Figure S2. UV absorbance spectra of samples for determination of conjugated diene values.

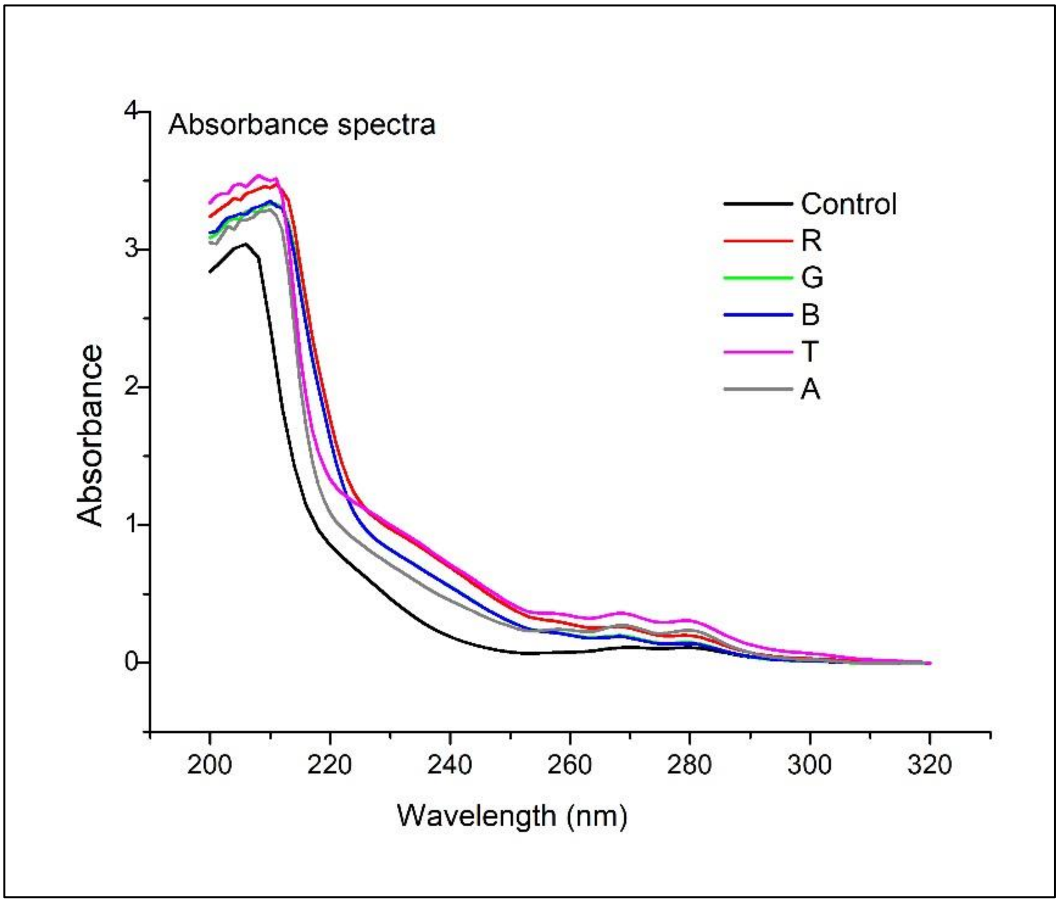

Figure S3. Transmittance spectra of Bandpass filters (BP) used for separation of emission signals in fluorescence lifetime measurements. (a)BP 460; (b) BP620.

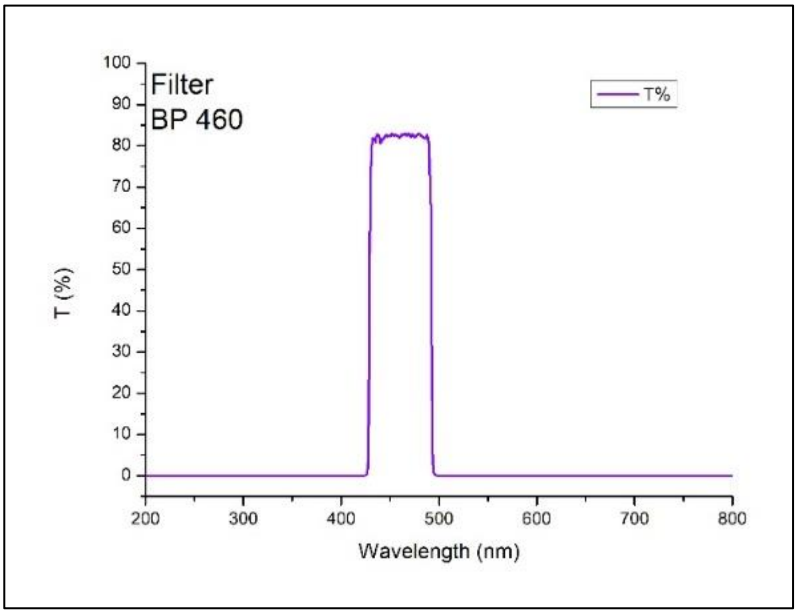


(a)

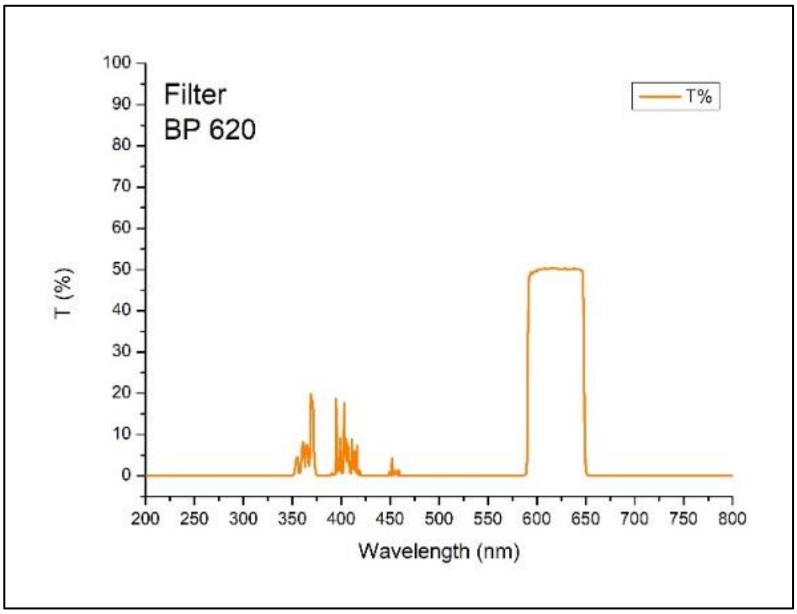


(b)

Figure S4 – Samples after the reaction time with the ready reagent supplied by the manufacturer for analysis of peroxides.(a) Control; (b) Sample R; (c) Sample G; (d) Sample B; (e) Sample T; (f) Sample A; (g) from left to right samples: R, G, B, T, A.

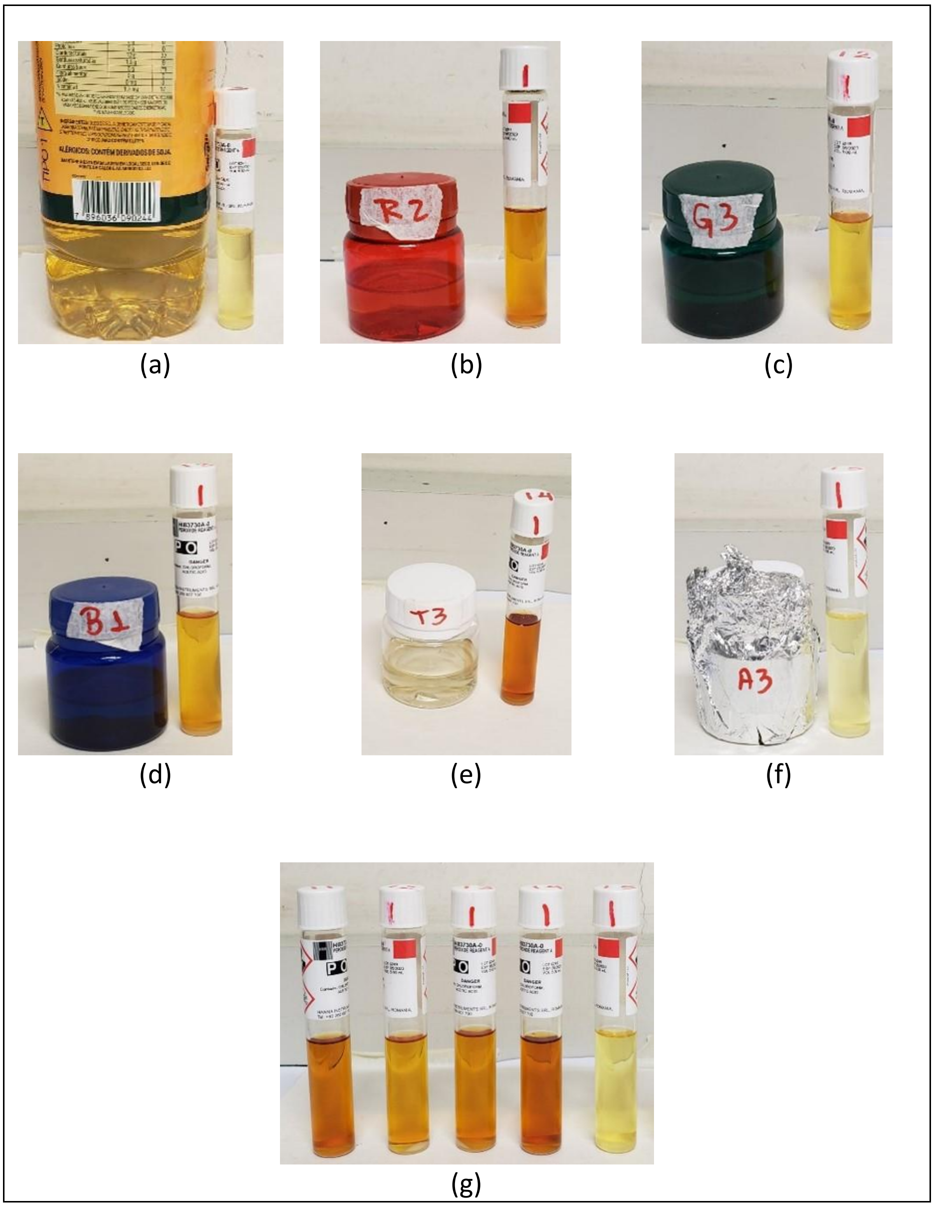

Supplement: Supplementary file 1 [file foods-12-01862-s001.zip › Figures S1-S4.pdf]
