# Supplementary material for: Evaluation of Steady-State and Time-Resolved Fluorescence Spectroscopy as a Method for Assessing the Impact of Photo-Oxidation on Refined Soybean Oils
Source: Foods. 2023 Apr 30;12(9):1862. doi: 10.3390/foods12091862 (PMC10178558; doi:10.3390/foods12091862)
Supplement: Supplementary file 1 [file foods-12-01862-s001.zip › Table S1.pdf]

“Evaluation of steady-state and time-resolved fluorescence spectroscopy as a method for assessing the impact of photo-oxidation on refined soybean oils”

Carla Regina Borges Lopes and Lilia Coronato Courrol

Supplementary Material

Table S1: Steady-state fluorescence parameters.

Emission Intensity I (CPS)

λEx / λEm.	373/448			373/659			405/468			405/659			500/547			500/659		
	I	St. Desv[1]	sample - control[2]	I	St. Desv[1]	sample - control[2]	I	St. Desv[1]	sample - control[2]	I	St. Desv[1]	sample - control[2]	I	St. Desv[1]	sample - control[2]	I	St. Desv[1]	sample - control[2]
Control	2.10E+06	---	---	3.46E+05	---	---	9.97E+05	---	---	1.22E+06	---	---	4.69E+04	---	---	1.35E+05		
G	1.93E+06	1.03E+05	-1.70E+05	3.44E+05	8.24E+03	-2.57E+03	8.78E+05	4.14E+04	-1.19E+05	1.21E+06	2.14E+04	-1.25E+04	1.86E+04	1.22E+03	-2.84E+04	1.45E+05	2.65E+03	1.04E+04
R	1.68E+06	4.76E+03	-4.15E+05	1.42E+05	5.13E+04	-2.04E+05	6.71E+05	2.78E+04	-3.26E+05	2.06E+05	8.79E+04	-1.02E+06	3.64E+04	2.51E+03	-1.05E+04	2.68E+04	3.40E+04	-1.08E+05
B	1.98E+06	6.01E+04	-1.13E+05	3.51E+05	1.51E+04	4.88E+03	6.54E+05	2.69E+04	-3.44E+05	1.29E+06	6.36E+04	6.45E+04	1.63E+04	1.06E+03	-3.07E+04	1.46E+05	9.25E+03	1.10E+04
T	1.86E+06	1.87E+06	-2.34E+05	6.37E+04	3.11E+04	-2.83E+05	4.35E+05	2.27E+04	-5.63E+05	1.35E+05	1.13E+03	-1.09E+06	7.85E+03	1.17E+03	-3.91E+04	1.75E+04	9.49E+03	-1.17E+05
A	2.27E+06	7.03E+04	1.77E+05	3.63E+05	7.71E+03	1.64E+04	1.00E+06	7.94E+03	3.01E+03	1.28E+06	4.38E+04	5.38E+04	4.41E+04	5.70E+02	-2.89E+03	1.40E+05	3.83E+03	5.31E+03

Area

λEx / λEm.	373/448			373/659			405/468			405/659			500/547			500/659		
	area	St. Desv[1]	sample - control[2]	area	St. Desv[1]	sample - control[2]	area	St. Desv[1]	sample - control[2]	area	St. Desv[1]	sample - control[2]	area	St. Desv[1]	sample - control[2]	area	St. Desv[1]	sample - control[2]
Control	2.42E+08	--	--	9.64E+06	--	--	1.01E+08	--	--	3.79E+07	--	--	3.09E+06	--	--	3.73E+06	--	--
G	2.17E+08	1.30E+07	-2.41E+07	1.03E+07	3.46E+05	6.73E+05	8.78E+07	4.08E+06	-1.35E+07	3.82E+07	3.00E+05	3.13E+05	1.17E+06	8.05E+04	-1.93E+06	4.27E+06	2.05E+04	5.36E+05
R	1.87E+08	8.03E+05	-5.42E+07	3.98E+06	1.69E+06	-5.66E+06	6.84E+07	3.54E+06	-3.28E+07	5.53E+06	3.19E+06	-3.24E+07	2.63E+06	3.03E+05	-4.64E+05	662612.564	1.06E+06	-3.07E+06
B	2.10E+08	3735228.641	-3.16E+07	1.04E+07	540755.1849	7.37E+05	5.90E+07	2.06E+06	-4.22E+07	4.16E+07	2.13E+06	3.71E+06	1.07E+06	5.91E+04	-2.02E+06	4.36E+06	3.47E+05	6.27E+05
T	1.88E+08	1.02E+06	-5.40E+07	1.65E+06	8.14E+05	-7.99E+06	3.79E+07	2.57E+06	-6.33E+07	3.77E+06	2.57E+06	-3.42E+07	514989.0566	1.07E+05	-2.58E+06	492439.2337	3.07E+05	-3.24E+06
A	2.62E+08	8.23E+06	2.07E+07	1.01E+07	3.46E+05	5.07E+05	1.02E+08	4.93E+05	5.97E+05	3.96E+07	1.54E+06	1.70E+06	2.95E+06	4.95E+04	-1.40E+05	3.85E+06	1.41E+05	1.17E+05

λEm max (nn)

λEx / λEm.	373/448			373/659			405/468			405/659			500/547			500/659		
	λEm max	St. Desv[1]	sample - control[2]	λEm max	St. Desv[1]	sample - control[2]	λEm max	St. Desv[1]	sample - control[2]	λEm max	St. Desv[1]	sample - control[2]	λEm max	St. Desv[1]	sample - control[2]	λEm max	St. Desv[1]	sample - control[2]
Control	449.1			660.7			472.0			660.0			550.0			660.0		
G	443.1	1.4	-6.0	661.1	0.0	0.4	468.9	5.7	-3.1	659.8	0.0	-0.2	548.1	4.7	-1.9	659.0	0.6	-1.0
R	437.0	0.7	-12.1	664.0	4.2	3.3	462.9	6.2	-9.1	669.0	0.7	9.0	554.0	5.0	4.0	669.9	5.2	9.9
B	438.7	2.1	-10.4	660.2	1.0	-0.6	459.2	1.0	-12.8	659.8	0.6	-0.2	551.0	4.2	1.0	659.9	0.0	-0.1
T	432.3	0.7	-16.8	668.8	0.7	8.1	459.1	1.0	-12.9	669.0	1.5	9.0	546.0	7.1	-4.0	668.0	0.7	8.0
A	446.9	3.5	-2.1	661.1	0.0	0.4	472.0	1.2	0.0	659.9	0.0	-0.1	547.2	2.0	-2.8	659.9	0.6	-0.1

1: Standard desviation of triplicates.

2: result of subtracting the sample value minus the control value.
